# Good Preservation of Stromal Cells and No Apoptosis in Human Ovarian Tissue after Vitrification

**DOI:** 10.1155/2014/673537

**Published:** 2014-04-03

**Authors:** Raffaella Fabbri, Rossella Vicenti, Maria Macciocca, Gianandrea Pasquinelli, Roberto Paradisi, Cesare Battaglia, Nicola Antonio Martino, Stefano Venturoli

**Affiliations:** ^1^Gynecology and Pathophysiology of Human Reproduction Unit, DIMEC, S.Orsola-Malpighi Hospital, University of Bologna, Via Massarenti 13, 40138 Bologna, Italy; ^2^Clinical Pathology, DIMES, S.Orsola-Malpighi Hospital, University of Bologna, Via Massarenti 9, 40138 Bologna, Italy; ^3^Veterinary Clinics and Animal Productions Unit, Department of Emergency and Organ Transplantation (DETO), University of Bari Aldo Moro, Valenzano, 70010 Bari, Italy

## Abstract

The aim of this study was to develop a vitrification procedure for human ovarian tissue cryopreservation in order to better preserve the ovarian tissue. Large size samples of ovarian tissue retrieved from 15 female-to-male transgender subjects (18–38 years) were vitrified using two solutions (containing propylene glycol, ethylene glycol, and sucrose at different concentrations) in an open system. Light microscopy, transmission electron microscopy, and TUNEL assay were applied to evaluate the efficiency of the vitrification protocol. After vitrification/warming, light microscopy showed oocyte nucleus with slightly thickened chromatin and irregular shape, while granulosa and stromal cells appeared well preserved. Transmission electron microscopy showed oocytes with slightly irregular nuclear shape and finely dispersed chromatin. Clear vacuoles and alterations in cellular organelles were seen in the oocyte cytoplasm. Stromal cells had a moderately dispersed chromatin and homogeneous cytoplasm with slight vacuolization. TUNEL assay revealed the lack of apoptosis induction by vitrification in all ovarian cell types. In conclusion after vitrification/warming the stromal compartment maintained morphological and ultrastructural features similar to fresh tissue, while the oocyte cytoplasm was slightly damaged. Although these data are encouraging, further studies are necessary and essential to optimize vitrification procedure.

## 1. Introduction

Ovarian tissue cryopreservation and its storage offer the hope to prepubertal girls or fertile age women who suffer from benign or malignant disease and want to safeguard their ovarian function against the hazardous effects of surgery, chemotherapy, and radiotherapy.

Despite the encouraging results much can still be done to minimize tissue damage during the cryopreservation procedure. It is known that the cryopreservation procedure leads to a loss in the follicular pool when compared with fresh ovarian tissue [1]. This result is principally due to ice crystal formation during the cryopreservation procedures, which can have a deleterious effect on cellular interactions and cell membranes [2].

Cryopreservation of ovarian tissue is primarily performed by slow freezing/rapid thawing for human fertility preservation and 24 live births and 4 ongoing pregnancies have been obtained after transplantation with this technique [3]. However, many studies have emphasized that the slow freezing of ovarian tissue safeguards the smallest follicles in the tissue, while it determined the greatest damage in the stroma cells [4, 5].

An emerging alternative procedure for the cryopreservation of ovarian tissue is represented by vitrification/warming. The vitrification is an ultrarapid cooling process that produces a glass-like solidification of cells by extreme elevation in viscosity, so as to avoid cellular injury caused by ice crystal formation [6–10]. In order to achieve successful vitrification, high cooling rates, as well as high concentrations of cryoprotectants are required.

This method has been successfully applied to preserve human blastocyst and oocyte [11, 12]. For ovarian tissue, good results have been reported in rodents, domestic animals, nonhuman primates, and human; even if, data on human ovarian tissue vitrification are still limited [13].

The aim of this study was to develop a vitrification procedure for human ovarian tissue cryopreservation in order to better preserve the ovarian tissue.

## 2. Materials and Methods

### 2.1. Patients

The study was conducted in 15 female-to-male transgender subjects (FTMs), 18–38 years, (28.19 ± 5.51, mean age ± standard deviation) suffering from gender identity disorder and undergoing sex reassignment surgery by hysterectomy-ovariectomy (Clinical trial n°61/2007/O/Tess) at Gynecology and Pathophysiology of Human Reproduction Unit, University of Bologna, Italy. The patients have donated their ovarian tissue for research. For each patient ovarian tissue samples were analyzed at the time of sample collection (fresh tissue, t0) and after vitrification/warming (vitrified/warmed tissue, t1). t1 sample was compared with t0 sample of the same patient to minimize the interpatient variation.

### 2.2. Tissue Sampling

For each patient, a bioptic ovarian sample was collected at the time of surgery and immediately transferred to the laboratory in a Dulbecco's phosphate buffered solution (PBS) (Gibco, Life Technologies LTD, Paisley, Scotland) with 10% inactivated human serum (provided by the Transfusion Centre of S.Orsola-Malpighi Hospital of Bologna, Italy). Bioptic ovarian sample was cut into slices 1,5 × 0,5 × 0,2 cm with a scalpel blade, placed in precooled plastic cryovials (Intermed Nunc Cryotubes, Roskilde, Denmark) and cryopreserved using the vitrification/warming protocol. For each patient, three samples (3 mm^2^) were processed for light microscopy, transmission electron microscopy, and TUNEL assay (fresh tissue, t0).

### 2.3. Vitrification/Warming Protocol

#### 2.3.1. Vitrification Protocol

The vitrification protocol was based on a two-step method in an open carrier. Each ovarian sample was placed in a cryovial containing 1.8 mL of equilibration solution consisting of 2 M propylene glycol (Fluka Chemical, Sigma Aldrich, SrL, Milan, Italy) + 3 M ethylene glycol (Fluka Chemical, Sigma Aldrich, SrL, Milan, Italy) + 0.2 M sucrose (Fluka Chemical, Sigma Aldrich, SrL, Milan, Italy) + 15% human serum in PBS and was transferred to a rolling system at 4°C for 30 min. Subsequently, the sample was transferred into a second cryovial containing 1.8 mL of vitrification solution consisting of 3 M propylene glycol + 5 M ethylene glycol + 0.5 M sucrose + 15% human serum in PBS and newly put onto a rolling system at 4°C for 30 min. Then, the sample was loaded in 200 *μ*L of vitrification solution on an open plastic support and quickly immersed into liquid nitrogen decontaminated according to Parmegiani protocol [14]. Following, the support containing the sample was placed in an empty cryovial (Intermed Nunc Cryotubes, Denmark) and stored into a liquid nitrogen tank for at least 1-2 months before warming.

#### 2.3.2. Warming Protocol

The support containing the sample was released from the cryovial, exposed at room temperature for 30 sec, and then immersed in a warming solution consisting of 1 M sucrose + 15% human serum in PBS at 39°C for 1 min, gently shaking until the ice was completely melted. Subsequently, the cryoprotectant was removed at 4°C by stepwise dilution of sucrose. The sample was transferred to the solution 1 (0.5 M sucrose + PBS + 15% human serum) for 3 min, and then to the solution 2 (0.25 M sucrose + 15% human serum in PBS) for 3 min. Finally, the warmed sample was rinsed in preequilibrated *α* minimal essential medium (*α*-MEM, Sigma Aldrich SrL; Milan, Italy) supplemented with penicillin/streptomycin (0.1 mg/mL) + 10% human serum two times at 4°C for 1 min. For each patient, warmed sample was fixed for the same analysis conducted on fresh sample (warmed tissue, t1).

### 2.4. Light and Transmission Electron Microscopy

For each patient, fresh and vitrified/warmed samples were fixed in a 4% paraformaldehyde solution in distilled water maintained at pH 7.4 overnight at 4°C. After osmium tetroxide postfixation and alcohol dehydration, the samples were embedded in epoxy resin (Araldite M hardener, Fluka, Buchs, Switzerland) and then sectioned using an ultramicrotome (Ultracut, Reichert, Vienna, Austria). For each sample, one 0.5 *μ*m thick section out of every 50 was collected and stained with toluidine blue. Sections were initially observed under a microscope (Nikon) at ×10 magnification to detect artifacts and discharge samples without cortical tissue. Sections were then observed at ×25 to establish the development stage of the follicle, according to Gougeon classification [15], and to evaluate architecture and nuclear and cytoplasm features of stromal cells. Finally, sections were analysed at ×40 to evaluate follicle and interstitial injury on at least 10 random microscopic high power fields (HPF) and scored using the method previously described by Fabbri et al. [5]. Semithin sections were viewed in a blind fashion by two different pathologists using a Leitz Diaplan light microscope equipped with a CCD JVC video camera, and digitized images were analyzed with Image ProPlus software.

After semithin sectioning, the 60 nm thick sections were collected on 200 mesh grids, stained with uranyl acetate followed by lead citrate and viewed using a Philips 410 T transmission electron microscope at 80 kV, in order to evaluate the ultrastructural features of follicles before and after vitrification/warming. Chromatin pattern, integrity of organelles, and membranes were carefully screened for oocytes, granulosa as well as stromal cells, according to previously reported subcellular criteria [5].

### 2.5. Apoptosis Analysis

For each patient, fresh and vitrified/warmed samples were fixed in 4% formaldehyde, embedded in paraffin, and serially sectioned at a thickness of 4 *μ*m. Every 5 slides, one was stained with haematoxylin and eosin (Merck KGaA, Darmstadt, Germany) for histological evaluation; the other slides were TUNEL stained, for apoptosis evaluation (POD, Roche, Mannheim, Germany), according to the manufacturer's instructions. Brown nuclei indicated TUNEL-positive cells. Follicles with more than two TUNEL-positive granulosa cells were considered to be apoptotic, as well as those follicles with TUNEL-positive oocytes. A total of 100 follicles were analyzed for each experimental condition. The number of apoptotic stromal cells was analyzed in a 100 *μ*m^2^ square (randomly assigned in the sections) and was counted three times and then averaged. The percentage of apoptotic cells was calculated as follows: apoptotic cell number/total cell number × 100 and quantified in a double blind fashion using a Leitz Diaplan light microscope equipped with a CCD JVC video camera. Digitized images were analyzed with Image ProPlus software.

### 2.6. Statistical Analysis

For each variable (follicle damage and interstitial edema), at least 10 fields were scored on an integer scale of 0–3. Differences in the mean score among fresh and vitrified/warmed samples were statistically analyzed using Kruskal-Wallis test followed by Dunn multiple comparison test (GraphPad Prism version 4). The Student's *t*-test was used to analyze the percentage of apoptotic cells observed in fresh and vitrified/warmed samples (GraphPad Prism version 4). *P* < 0.05 was considered statistically significant.

## 3. Results

### 3.1. Light and Transmission Electron Microscopy

A total of 1479 follicles (from fresh and vitrified/warmed samples) were analyzed by light microscopy to evaluate the morphology, the developmental stage, and the percentage of degenerated follicles on the total number of follicles (Table 1). The number of follicles was equally distributed among fresh and vitrified/warmed samples (53% versus 47%; *P* = 0.905). The follicle density (follicle number per mm^2^ of the overall section area) was 4.28 ± 6.15/mm^2^ in fresh and 4.12 ± 6.48/mm^2^ in vitrified/warmed tissue (*P* = 0.269). Overall, 1429 follicles (96.5%) were primordial and intermediary, 43 (3%) primary, 5 (0.36%) secondary, 1 (0.07%) preantral, and 1 (0.07%) antral.

**Figure 1:**
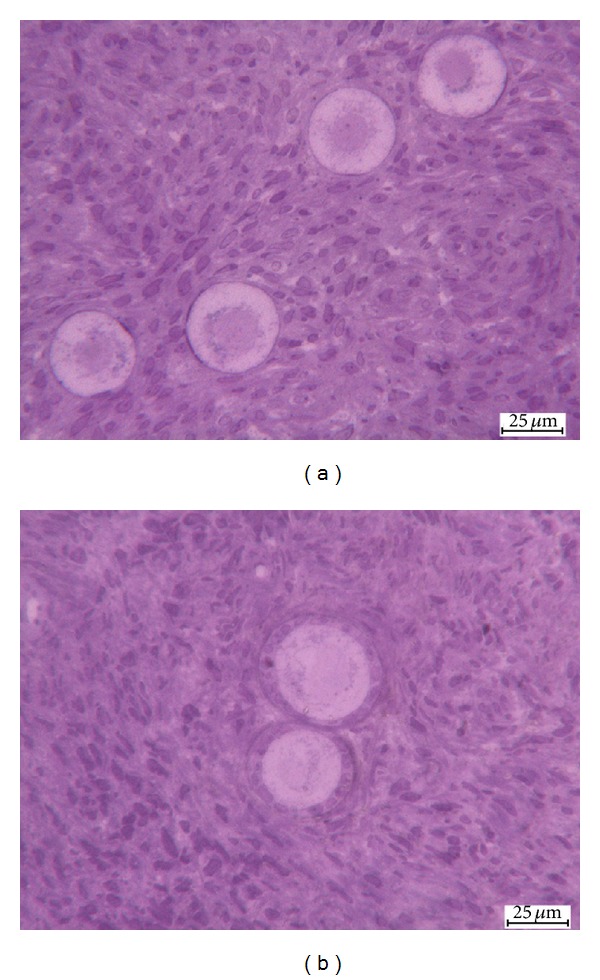
Light microscopy images of t0 (a) and t1 (b) samples. Well-preserved clusters of follicles displaying euchromatic nuclei and preserved oocyte cytoplasm. Compact stromal cells. t0 = fresh tissue and t1 = vitrified/warmed tissue.

In fresh samples follicles showed close adherence between oocyte and granulosa cells with normal morphological appearance: the oocytes had many mitochondria located around the nucleus, homogenous cytoplasm, and no vacuoles were observed. Stromal cells had oval- to spindle-shaped nucleus with a finely dispersed chromatin, small cytoplasm vacuoles were seen, and no interstitial edema was present (Figure 1(a)).

The vitrified samples showed oocyte nucleus with slightly thickened chromatin and irregular shape, while granulosa cells were well preserved. The architecture of the ovarian stromal was intact, maintaining the features of not vitrified tissue even though an increased cytoplasm vacuolization was observed (Figure 1(b)).

**Figure 2:**
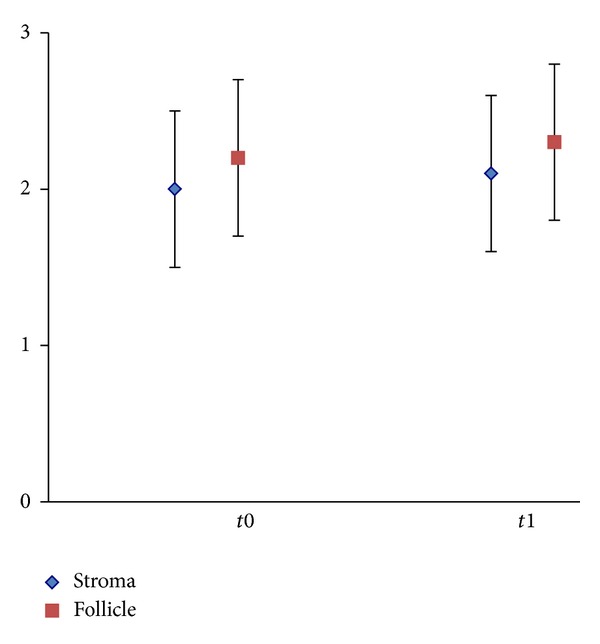
Statistical analysis, showing scores for follicle and stroma preservation in fresh (t0) and vitrified/warmed (t1) ovarian cortical tissue. Values are expressed as mean ± SD. Score interpretation: 0 = good preservation, 1 = poor preservation, 2 = bad preservation, and 3 = worst preservation. *P* = 0.17 for stroma and *P* = 0.53 for follicles.

The data obtained by statistical analysis showed that no significant differences were observed in the overall preservation of ovarian tissue processed according to the vitrification protocol when compared with fresh tissue (Figure 2).

**Figure 3:**
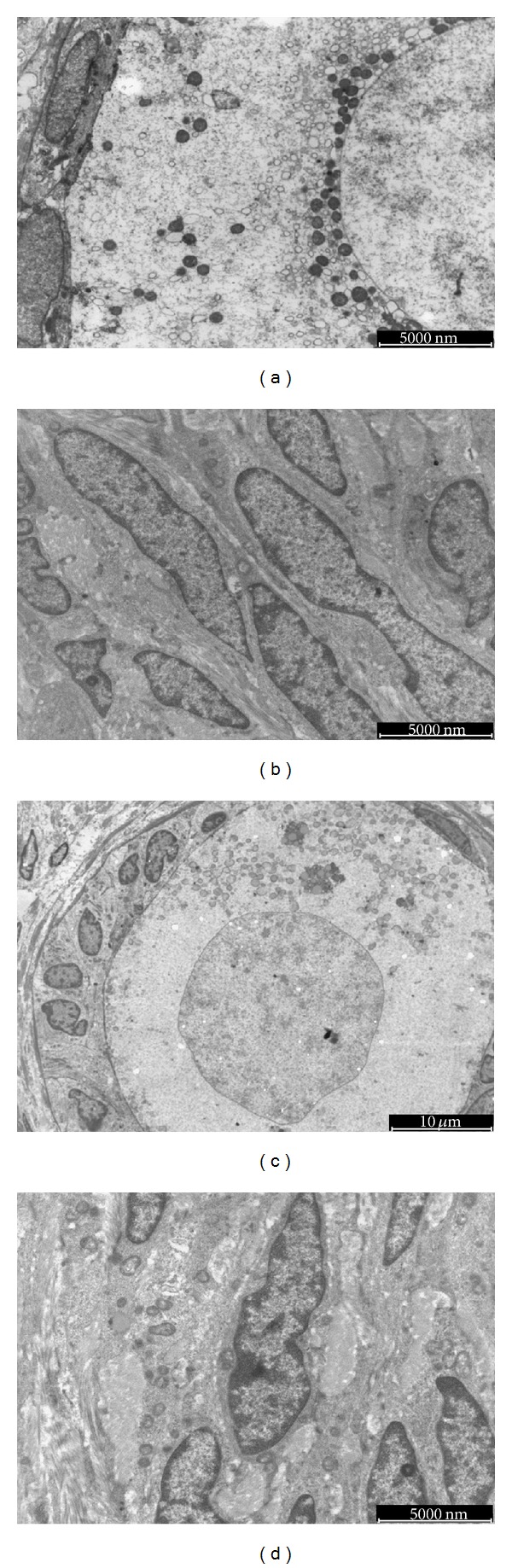
Ultrastructure images of t0 ((a), (b)) and t1 ((c), (d)) ovarian cortical tissue. A primordial follicle with a layer of flattened granulosa cells. The oocyte nucleus shows homogeneous euchromatin surrounded by the nuclear membrane. Cytoplasm is well organized with perinuclear round mitochondria (a). Spindle stromal cells with moderately dispersed chromatin and homogeneous cytoplasm (b). A primary follicle with a layer of cuboidal granulosa cell firmly attached to the oocyte. The oocyte nucleus has a slight irregular outline and euchromatin content. Mitochondria are eccentrically placed at one side of the oocyte cytoplasm (c). Stromal cells have moderately dispersed chromatin, compact cytoplasm with enlarged mitochondria (d). t0 = fresh tissue and t1 = vitrified/warmed tissue.

To extend and validate LM evaluation, ultrastructural analysis was performed. A total of 132 follicles, equally distributed among fresh and vitrified/warmed samples (70 versus 62, *P* = 0.712), were analyzed. The fresh samples showed follicles containing large oocytes with regular nuclei, finely dispersed chromatin, nuclear pores, and abundant cytoplasm. Mitochondria appeared typically rounded, with a low-density matrix and few peripheral cristae, clustered in clouds or arranged in a rosette-like pattern around a dense, amorphous or finely granular intermitochondrial substance. Golgi apparatus was well developed; a few cisternae of rough endoplasmic reticulum were seen and occasional lipid inclusions and lipofuscin bodies as well. A close adhesion between oocyte and follicular cells was commonly seen. Granulosa cells showed flat nuclei with one or two reticular nucleoli. Rod-shaped mitochondria, free ribosomes, rough and smooth endoplasmic reticulum, and scattered vacuoles were observed in the cell cytoplasm (Figure 3(a)). The stroma showed spindle cell with moderately dispersed chromatin and homogeneous cytoplasm and slight signs of vacuolization (Figure 3(b)).

The vitrified/warmed follicles had nucleus with slight irregular shape and finely dispersed chromatin. Clear vacuoles were seen in the oocyte cytoplasm and a slight clarification of the cytoplasmic matrix as well. Cytoplasmic organelles were altered in shape and distribution. Irregularly shaped or swollen mitochondria were found scattered in the cytoplasm or around large vacuoles. Granulosa cells were adherent to oocyte (Figure 3(c)). Stromal cells showed moderately dispersed chromatin and homogeneous cytoplasm with slight vacuolization and enlarged mitochondria (Figure 3(d)).

### 3.2. TUNEL Assay

**Figure 4:**
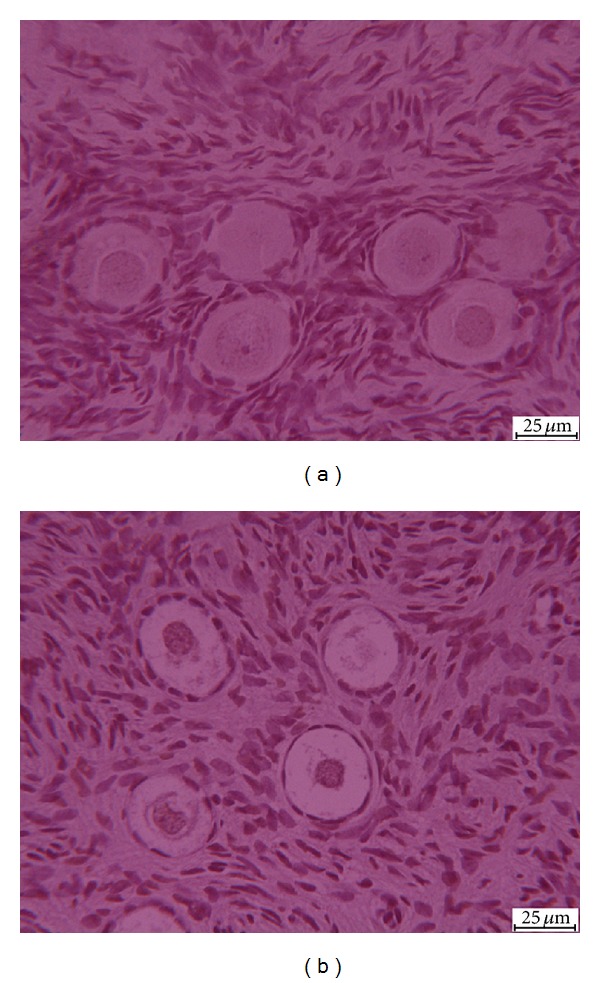
TUNEL images of t0 (a) and t1 (b) samples. t0 = fresh tissue and t1 = vitrified/warmed tissue.

Only follicles with a visible oocyte nucleus were analyzed. The percentage of apoptotic follicles was 5% and 6% in fresh and vitrified/warmed samples, respectively (*P* = 0.13). The percentage of DNA damaged stromal cells evaluated in fresh and vitrified/warmed samples was 11% and 12%, respectively (*P* = 0.18; Figure 4).

## 4. Discussion 

Today the cryopreservation of ovarian tissue before the start of cancer treatments is proposed as an option for preserving fertility. It allows recovering of a large number of follicles; it may be performed at any time of the menstrual cycle avoiding delay in the onset of antineoplastic therapy; it is particularly indicated in patients with hormone-related cancers, and it is the only option for prepubertal patients [3].

The critical points in the cryopreservation of ovarian tissue are determined by the presence of heterogeneous cellular components (oocytes, granulosa, and stromal cells) with different properties, which make the osmotic dehydration and rehydration extremely variable [16].

Cryopreservation strategies for ovarian tissue have therefore aimed to optimize methods and protocols for the preservation of whole tissue [13].

In the last decade there has been a great interest for the vitrification, since it might prevent tissue damage due to ice crystal formation. The success of vitrification depends on many factors such as sample size, type and concentration of cryoprotectants, temperature of exposure to vitrification solution, stepwise addition of vitrification solution, carrier system, quality of samples, and technical expertise [13].

Because of so many variables, the results obtained from vitrification are discrepant from species to species and within the same species [13].

The vitrification of ovarian tissue in animal models (mouse, sheep, ovine, bovine, monkey, hamster, rabbit, and baboon) has produced promising results: follicles maintain the same survival rates, morphology, and ultrastructure comparable to fresh follicles, yielding even live births in some species [3, 13].

In human, many studies support vitrification as the method of choice for ovarian tissue cryopreservation, providing similar results to conventional freezing, with the additional advantage of preserving the ultrastructure of stromal tissue that is usually affected by freezing [1, 2, 13, 17–19]. However the live birth of babies from transplantation of vitrified ovarian tissue has not yet been obtained. Therefore, despite the encouraging results, absolute conclusions cannot easily be drawn.

In our study large size samples and two solutions were used to vitrify human ovarian tissue in an open system. In order to promote the dehydration of large size samples, a combination of ethylene glycol, propylene glycol, and sucrose at different concentrations and a long equilibration time were applied. Light and transmission electron microscopy and TUNEL analysis were applied to monitor the efficiency of our vitrification protocol.

The literature data show that the sample's sizes have a strong influence on the preservation; in fact using small specimens with better results has been obtained [13]. However studies in sheep by Bordes group [20] obtained the birth of four lambs and the recovery of ovarian endocrine function by half ovary vitrification [20, 21]. In addition, data reported by Ferreira et al. [22] on bovine ovarian cortex showed that there was an increased risk of morphological damage to primary and primordial follicles when the tissue slices were cut with all dimension larger than 2 mm, provided that one of the dimensions of the fragment slice is maintained ≤2 mm [22]. The vitrification of large size samples of human ovarian tissue could be particularly useful, because the handling of the tissue and the gain in terms of follicular population are better using the larger samples [23].

Our results show that after vitrification/warming the stromal compartment maintains morphological and ultrastructural features similar to those seen in fresh tissue. Regarding follicles, the granulosa cells appear well preserved, while the oocyte appears slightly negatively affected by vitrification, as shown by increased cytoplasm vacuolization and organelle changes. These features might be considered as early signs of follicular degeneration, likely due to an inadequate dehydration and/or osmotic stress [24].

Our results are in agreement with those reported by other authors [25–28]. Gandolfi et al. [25] showed that vitrification caused extensive morphological damage to primordial follicle in human ovarian tissue. Isachenko et al. [26] compared conventional freezing and vitrification of human ovarian tissue in terms of follicular quality, steroidogenic activity, and proliferative potential by GAPDH gene expression. The results provided evidence that conventional freezing allowed a better preservation of all types of follicles and a higher proliferative capacity of the tissue than vitrification, concluding that conventional freezing is the method of choice for the cryopreservation of human ovarian tissue. Irregularly shaped or swollen mitochondria scattered in the cytoplasm of both oocytes and follicular cells were reported by Zhou et al. [27] using conventional vitrification. Oktem et al. [28] reported that vitrified ovaries contained statistically significantly fewer primordial follicles and reported statistically significantly less hormonal production in vitro when compared with fresh and slow-frozen ovaries.

In the present study we also evaluated the frequency of apoptosis in ovarian tissue after vitrification. In agreement with previous studies, TUNEL assay does not show evidence of any statistical difference in apoptosis between fresh and vitrified samples [17, 18]. This finding suggests that the cryodamages induced by vitrification occur mainly in cytoplasmic organelles and cell membranes, preserving the nuclear compartment. In fact, at nuclear level the most likely consequence of vitrification would be the necrosis process, which can be better demonstrated using careful morphological and ultrastructural analysis [16].

Certainly a limitation of the present study has been the lack of functional tests that allowed determining if the damage done to oocytes was reversible and transitory or not. Further investigation such as xeno-transplantation might give information about viability of the follicle within the tissue. Nevertheless, tissue culture might be an excellent alternative method to demonstrate the viability of the tissue.

## 5. Conclusions

In conclusion, our vitrification protocol showed a good preservation of the stromal compartment, as also reported by other authors in the literature. This finding indicates that vitrification protocols are efficient for human ovarian tissue cryopreservation compared with conventional freezing, because stromal damages are avoided. However, in our study slight damages to the oocytes were observed after vitrification. Therefore, further studies are necessary and essential to optimize the vitrification protocol and to decide to switch from conventional freezing to vitrification for human ovarian tissue cryopreservation. The final proof to propose vitrification of ovarian tissue to patients will only be the birth of healthy babies after reimplantation of vitrified tissue.

## Figures and Tables

**Table 1:** Number and % of degenerated follicles on the total number of follicles parted for primordial, primary, secondary, preantral, and antral stage.

Samples (*n* = 15)	Degenerated follicles/total number of follicles (%)					
	Primordial/intermediary	Primary	Secondary	Preantral	Antral	TOT.
t0	215/739 (29)	4/15 (27)	1/2 (50)	0/1 (0%)	0/0 (0%)	757
t1	221/690 (32)	9/28 (32)	1/3 (33)	0/0 (0%)	0/1 (0%)	722

t0: fresh tissue; t1: vitrified/warmed tissue.
